# Dipyridinium 2,2′-dithio­dinicotinate

**DOI:** 10.1107/S1600536809013543

**Published:** 2009-04-18

**Authors:** Wagee A. Yehye, Azhar Ariffin, Noorsaadah Abdul Rahman, Seik Weng Ng

**Affiliations:** a Department of Chemistry, University of Malaya, 50603 Kuala Lumpur, Malaysia

## Abstract

The dianion of the title salt, 2C_5_H_6_N^+^·C_12_H_6_N_2_O_4_S_2_^2−^, lies on a special position of 2 site symmetry that relates one thio­nicotinate part to the other, and the dihedral angle between the niotinate planes is 89.2 (2)°. The pyridinium cations are hydrogen bonded to the carboxyl­ate group by way of N—H⋯O links.

## Related literature

The structure is a non-merohedral twin; for the program to model twinned crystal structures, see: Spek (2003 ▶). For 1,1′-dithio-2,2′-dinicotinic acid, see: Zhu *et al.* (2002 ▶). For the methyl, ethyl and *n*-butyl esters, see: Cindrić *et al.* (2001 ▶); Toma *et al.* (2004 ▶).
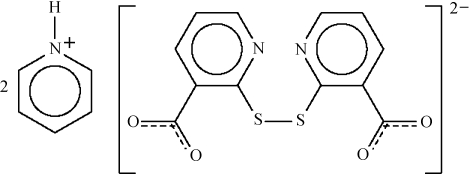


## Experimental

### 

#### Crystal data

2C_5_H_6_N^+^·C_12_H_6_N_2_O_4_S_2_^2−^
*M* *_r_* = 466.52
Monoclinic, 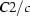

*a* = 7.9621 (3) Å
*b* = 12.3354 (4) Å
*c* = 21.5057 (8) Å
β = 95.917 (2)°
*V* = 2100.9 (1) Å^3^
*Z* = 4
Mo *K*α radiation
μ = 0.29 mm^−1^
*T* = 123 K
0.28 × 0.16 × 0.08 mm

#### Data collection

Bruker SMART APEX diffractometer
Absorption correction: multi-scan (*SADABS*; Sheldrick, 1996 ▶) *T*_min_ = 0.923, *T*_max_ = 0.977
6726 measured reflections
1855 independent reflections
1496 reflections with *I* > 2σ(*I*)
*R*_int_ = 0.101

#### Refinement

*R*[*F* ^2^ > 2σ(*F* ^2^)] = 0.092
*wR*(*F* ^2^) = 0.269
*S* = 1.59
1855 reflections
146 parameters
H-atom parameters constrained
Δρ_max_ = 0.47 e Å^−3^
Δρ_min_ = −0.57 e Å^−3^

### 

Data collection: *APEX2* (Bruker, 2008 ▶); cell refinement: *SAINT* (Bruker, 2008 ▶); data reduction: *SAINT*; program(s) used to solve structure: *SHELXS97* (Sheldrick, 2008 ▶); program(s) used to refine structure: *SHELXL97* (Sheldrick, 2008 ▶); molecular graphics: *X-SEED* (Barbour, 2001 ▶); software used to prepare material for publication: *publCIF* (Westrip, 2009 ▶).

## Supplementary Material

Crystal structure: contains datablocks global, I. DOI: 10.1107/S1600536809013543/si2170sup1.cif

Structure factors: contains datablocks I. DOI: 10.1107/S1600536809013543/si2170Isup2.hkl

Additional supplementary materials:  crystallographic information; 3D view; checkCIF report

## Figures and Tables

**Table 1:** Hydrogen-bond geometry (Å, °)

*D*—H⋯*A*	*D*—H	H⋯*A*	*D*⋯*A*	*D*—H⋯*A*
N2—H2⋯O2	0.88	1.71	2.586 (7)	174

**Fig. 1.**
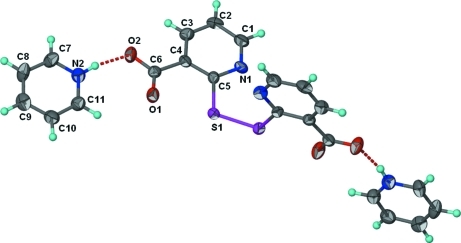
Thermal ellipsoid plot (Barbour, 2001) of 2(C5H6N) (C12H6N2O4S2) at the 70% probability level. Hydrogen atoms are drawn as spheres of arbitrary radius.

## supplementary crystallographic information

### Experimental

The title compound was isolated as one of the by-products when 2-(3,5-di-*tert*-butyl-4-hydroxybenzylsulfanyl)nicotinic acid (0.37 g, 1 mmol) and thiocarbohydrazide (0.10 g, 1 mmol) were reacted in pyridine (10 ml) for 3 h. The product from a cool mixture was collected and recrystallized from pyridine

### Refinement

The specimen used in the diffraction measurements is a multiply-twinned crystal; twinning was evident when examined by the RLATT routine of the data collection software, with a major of about 60%. The diffraction images were integrated on the major component.

The structure initially refined to an *R>* index of 13%. The structure is a non-merohedral twin, as suggested by *PLATON* (Spek, 2003). The intensities were de-twinned by the *TwinRotMat* routine.

The carbon- and nitrogen-bound H-atoms were placed in calculated positions (C—H 0.95 Å, N–H 0.88 Å) and were included in the refinement in the riding model approximation, with *U*(H) set to 1.2 times *U*(C,*N*).

### Crystal data

**Table:**

2C_5_H_6_N^+^·C_12_H_6_N_2_O_4_S_2_^2^^−^	*F*(000) = 968
*M**_r_* = 466.52	*D*_x_ = 1.475 Mg m^−^^3^
Monoclinic, *C*2/*c*	Mo *K*α radiation, λ = 0.71073 Å
Hall symbol: -C 2yc	Cell parameters from 1585 reflections
*a* = 7.9621 (3) Å	θ = 3.1–24.0°
*b* = 12.3354 (4) Å	µ = 0.29 mm^−^^1^
*c* = 21.5057 (8) Å	*T* = 123 K
β = 95.917 (2)°	Chip, light yellow
*V* = 2100.9 (1) Å^3^	0.28 × 0.16 × 0.08 mm
*Z* = 4	

### Data collection

**Table:**

Bruker SMART APEX diffractometer	1855 independent reflections
Radiation source: fine-focus sealed tube	1496 reflections with *I* > 2σ(*I*)
graphite	*R*_int_ = 0.101
ω scans	θ_max_ = 25.0°, θ_min_ = 1.9°
Absorption correction: multi-scan (*SADABS*; Sheldrick, 1996)	*h* = −9→9
*T*_min_ = 0.923, *T*_max_ = 0.977	*k* = −14→14
6726 measured reflections	*l* = −25→25

### Refinement

**Table:**

Refinement on *F*^2^	Primary atom site location: structure-invariant direct methods
Least-squares matrix: full	Secondary atom site location: difference Fourier map
*R*[*F*^2^ > 2σ(*F*^2^)] = 0.092	Hydrogen site location: inferred from neighbouring sites
*wR*(*F*^2^) = 0.269	H-atom parameters constrained
*S* = 1.59	*w* = 1/[σ^2^(*F*_o_^2^) + (0.1*P*)^2^ + 5*P*] where *P* = (*F*_o_^2^ + 2*F*_c_^2^)/3
1855 reflections	(Δ/σ)_max_ = 0.001
146 parameters	Δρ_max_ = 0.47 e Å^−^^3^
0 restraints	Δρ_min_ = −0.57 e Å^−^^3^

### Fractional atomic coordinates and isotropic or equivalent isotropic displacement parameters (Å^2^)

**Table:**

	*x*	*y*	*z*	*U*_iso_*/*U*_eq_	
S1	0.5434 (2)	0.61088 (12)	0.29627 (7)	0.0213 (5)	
O1	0.6450 (6)	0.6042 (4)	0.4204 (2)	0.0317 (12)	
O2	0.5265 (6)	0.6888 (4)	0.4968 (2)	0.0336 (12)	
N1	0.3073 (7)	0.7643 (4)	0.2874 (2)	0.0249 (12)	
N2	0.7396 (7)	0.5816 (4)	0.5721 (3)	0.0278 (13)	
H2	0.6690	0.6218	0.5478	0.033*	
C1	0.2040 (9)	0.8378 (5)	0.3103 (3)	0.0299 (16)	
H1	0.1300	0.8778	0.2814	0.036*	
C2	0.1994 (8)	0.8583 (5)	0.3731 (3)	0.0271 (15)	
H2A	0.1240	0.9102	0.3873	0.033*	
C3	0.3092 (8)	0.8001 (5)	0.4142 (3)	0.0249 (14)	
H3	0.3091	0.8116	0.4579	0.030*	
C4	0.4190 (8)	0.7255 (5)	0.3929 (3)	0.0204 (13)	
C5	0.4125 (8)	0.7097 (5)	0.3280 (3)	0.0191 (13)	
C6	0.5418 (8)	0.6678 (5)	0.4383 (3)	0.0227 (14)	
C7	0.7634 (9)	0.5987 (6)	0.6336 (3)	0.0311 (16)	
H7	0.7030	0.6557	0.6510	0.037*	
C8	0.8721 (9)	0.5368 (6)	0.6728 (3)	0.0342 (17)	
H8	0.8862	0.5512	0.7164	0.041*	
C9	0.9605 (9)	0.4535 (6)	0.6481 (3)	0.0325 (16)	
H9	1.0335	0.4081	0.6744	0.039*	
C10	0.9398 (10)	0.4379 (6)	0.5841 (3)	0.0329 (16)	
H10	1.0030	0.3842	0.5651	0.039*	
C11	0.8259 (9)	0.5018 (5)	0.5485 (3)	0.0280 (15)	
H11	0.8078	0.4883	0.5049	0.034*	

### Atomic displacement parameters (Å^2^)

**Table:**

	*U*^11^	*U*^22^	*U*^33^	*U*^12^	*U*^13^	*U*^23^
S1	0.0232 (9)	0.0189 (8)	0.0212 (8)	0.0044 (6)	−0.0002 (6)	0.0000 (6)
O1	0.035 (3)	0.036 (3)	0.023 (2)	0.016 (2)	−0.003 (2)	−0.0003 (19)
O2	0.038 (3)	0.040 (3)	0.022 (2)	0.017 (2)	−0.002 (2)	0.000 (2)
N1	0.023 (3)	0.021 (3)	0.029 (3)	0.010 (2)	−0.003 (2)	0.000 (2)
N2	0.025 (3)	0.029 (3)	0.029 (3)	0.001 (2)	−0.003 (2)	0.000 (2)
C1	0.029 (4)	0.020 (3)	0.039 (4)	0.004 (3)	−0.003 (3)	0.001 (3)
C2	0.023 (3)	0.018 (3)	0.040 (4)	0.000 (3)	0.004 (3)	−0.003 (3)
C3	0.027 (3)	0.023 (3)	0.027 (3)	0.004 (3)	0.009 (3)	−0.001 (3)
C4	0.019 (3)	0.016 (3)	0.025 (3)	−0.003 (2)	−0.003 (3)	0.002 (2)
C5	0.020 (3)	0.018 (3)	0.019 (3)	0.000 (2)	0.003 (2)	0.003 (2)
C6	0.024 (3)	0.017 (3)	0.026 (3)	−0.001 (3)	−0.001 (3)	−0.001 (2)
C7	0.027 (4)	0.037 (4)	0.028 (4)	−0.002 (3)	−0.003 (3)	−0.006 (3)
C8	0.024 (4)	0.053 (5)	0.024 (3)	0.001 (3)	−0.005 (3)	−0.001 (3)
C9	0.021 (3)	0.037 (4)	0.038 (4)	−0.003 (3)	−0.005 (3)	0.004 (3)
C10	0.034 (4)	0.025 (3)	0.039 (4)	0.002 (3)	−0.002 (3)	−0.001 (3)
C11	0.028 (4)	0.029 (3)	0.027 (3)	0.002 (3)	0.002 (3)	−0.002 (3)

### Geometric parameters (Å, °)

**Table:**

S1—C5	1.785 (6)	C3—C4	1.380 (9)
S1—S1^i^	2.038 (3)	C3—H3	0.9500
O1—C6	1.226 (8)	C4—C5	1.405 (9)
O2—C6	1.302 (8)	C4—C6	1.490 (8)
N1—C5	1.329 (8)	C7—C8	1.375 (10)
N1—C1	1.350 (8)	C7—H7	0.9500
N2—C11	1.330 (9)	C8—C9	1.383 (10)
N2—C7	1.333 (8)	C8—H8	0.9500
N2—H2	0.8800	C9—C10	1.384 (10)
C1—C2	1.376 (9)	C9—H9	0.9500
C1—H1	0.9500	C10—C11	1.372 (10)
C2—C3	1.380 (9)	C10—H10	0.9500
C2—H2A	0.9500	C11—H11	0.9500
			
C5—S1—S1^i^	102.7 (2)	C4—C5—S1	120.8 (5)
C5—N1—C1	117.8 (6)	O1—C6—O2	124.2 (6)
C11—N2—C7	117.9 (6)	O1—C6—C4	121.1 (6)
C11—N2—H2	121.0	O2—C6—C4	114.7 (5)
C7—N2—H2	121.0	N2—C7—C8	122.4 (7)
N1—C1—C2	124.0 (6)	N2—C7—H7	118.8
N1—C1—H1	118.0	C8—C7—H7	118.8
C2—C1—H1	118.0	C7—C8—C9	119.4 (7)
C1—C2—C3	117.0 (6)	C7—C8—H8	120.3
C1—C2—H2A	121.5	C9—C8—H8	120.3
C3—C2—H2A	121.5	C8—C9—C10	118.2 (6)
C4—C3—C2	121.0 (6)	C8—C9—H9	120.9
C4—C3—H3	119.5	C10—C9—H9	120.9
C2—C3—H3	119.5	C11—C10—C9	118.6 (7)
C3—C4—C5	117.6 (6)	C11—C10—H10	120.7
C3—C4—C6	119.8 (6)	C9—C10—H10	120.7
C5—C4—C6	122.6 (6)	N2—C11—C10	123.4 (6)
N1—C5—C4	122.5 (6)	N2—C11—H11	118.3
N1—C5—S1	116.7 (5)	C10—C11—H11	118.3
			
C5—N1—C1—C2	−1.0 (10)	S1^i^—S1—C5—C4	172.6 (5)
N1—C1—C2—C3	0.6 (10)	C3—C4—C6—O1	−177.2 (6)
C1—C2—C3—C4	0.5 (9)	C5—C4—C6—O1	0.9 (9)
C2—C3—C4—C5	−1.2 (9)	C3—C4—C6—O2	4.3 (8)
C2—C3—C4—C6	177.1 (6)	C5—C4—C6—O2	−177.5 (6)
C1—N1—C5—C4	0.2 (9)	C11—N2—C7—C8	−0.4 (10)
C1—N1—C5—S1	178.5 (5)	N2—C7—C8—C9	−0.1 (11)
C3—C4—C5—N1	0.9 (9)	C7—C8—C9—C10	2.1 (11)
C6—C4—C5—N1	−177.3 (5)	C8—C9—C10—C11	−3.5 (10)
C3—C4—C5—S1	−177.4 (5)	C7—N2—C11—C10	−1.2 (10)
C6—C4—C5—S1	4.4 (8)	C9—C10—C11—N2	3.2 (11)
S1^i^—S1—C5—N1	−5.7 (5)		

Symmetry codes: (i) −*x*+1, *y*, −*z*+1/2.

### Hydrogen-bond geometry (Å, °)

**Table:**

*D*—H···*A*	*D*—H	H···*A*	*D*···*A*	*D*—H···*A*
N2—H2···O2	0.88	1.71	2.586 (7)	174
